# Capturing the most wanted taxa through cross-sample correlations

**DOI:** 10.1038/ismej.2016.35

**Published:** 2016-03-04

**Authors:** Mathieu Almeida, Mihai Pop, Emmanuelle Le Chatelier, Edi Prifti, Nicolas Pons, Amine Ghozlane, S Dusko Ehrlich

**Affiliations:** 1 Center for Bioinformatics and Computational Biology, University of Maryland, College Park, MD, USA; 2 Department of Computer Science, University of Maryland, College Park, MD, USA; 3 INRA, Institut National de la Recherche Agronomique, US 1367 Metagenopolis, Jouy en Josas, France

## Abstract

The Human Microbiome Project (HMP) identified the 16S rRNA gene sequences of 'most wanted' taxa—prevalent in the healthy human microbiota but distant from previously known sequences. Since 2012, few of the corresponding genomes have been isolated and sequenced, and only through advanced isolation techniques. We demonstrate that the genomes of the most wanted taxa can be identified computationally through their correlation in abundance across multiple public metagenomic data sets. We link over 200 most wanted sequences with nearly complete genome sequences, including half of the taxa identified as high-priority targets by the HMP. The genomes we identify have strong similarity to genomes reconstructed through expensive isolation techniques, and provide a more complete functional characterization of these organisms than can be extrapolated from their 16S rRNA gene. We also provide insights into the function of organisms for which 16S rRNA gene signatures were recently reported to be associated with health and host genetic factors.

## Introduction

The majority of organisms inhabiting our bodies and our world cannot be readily cultured. As a result, most microbial communities can only be explored through culture-independent molecular techniques. Among these, two major approaches dominate: PCR-based amplification and sequencing of hypervariable regions of the 16S rRNA gene, and random sequencing of whole-community DNA. The former approach was pioneered in the 1980s by Norm Pace (Olsen *et al.*, 1986), and has resulted in the creation of an extensive catalog of 16S rRNA sequences comprising today over two million distinct entries. These data are widely available through three main databases: RDP (Cole *et al.*, 2009), Silva (Quast *et al.*, 2013) and Greengenes (DeSantis *et al.*, 2006). The random sequencing of community DNA is being increasingly applied to the study of environmental (Tyson *et al.*, 2004; Rusch *et al.*, 2007) and human-associated microbial communities (Gill *et al.*, 2006; Qin *et al.*, 2010; Human Microbiome Project C, 2012).

Unlike 16S rRNA-based studies, whole-community metagenomic projects suffer from the relative dearth of extensive and broad reference databases as the genomic data in public databases (in terms of both genes and genomes) is primarily derived from the relatively small number of genomes that have been grown in culture. The public data are also biased in their phylogenetic distribution (Wu *et al.*, 2009). Recent studies have begun to develop new reference catalogs based on the genes and genomes found in environmental (Yooseph *et al.*, 2007) and host-associated (Qin *et al.*, 2010; Human Microbiome Project C, 2012) metagenomic data sets and have already added millions of new genes to public databases such as MG-RAST (Wilke *et al.*, 2016) and IMG/M (Markowitz *et al.*, 2008). Missing from these catalogs are reliable connections to the wealth of information already accumulated in 16S rRNA studies. Creating a link between 16S rRNA amplicon studies and whole-community metagenomic data is complicated by the fact that the 16S rRNA gene is frequently misassembled or left unassembled even in isolate genome assemblies, let alone metagenomic mixtures. A testament to this difficulty are extensive efforts undertaken during the past couple of years to isolate or culture the organisms associated with the ‘most wanted' 16S rRNA gene sequences—sequences without close relatives in public databases yet found in high abundance in multiple samples from the HMP project (Fodor *et al.*, 2012). To date, just two of these organisms have been isolated and sequenced (Ma *et al.*, 2014; Rettedal *et al.*, 2014). Here we demonstrate that *in silico* analyses of the abundance of genomic entities (genes and 16S rRNA Operational Taxonomic Units (OTUs)) are effective in identifying links between 16S rRNA sequences and their genomic context. We specifically provide a first link between metagenomic species developed in the MetaHIT project (Qin *et al.*, 2010) and the data generated by the HMP project (Human Microbiome Project C, 2012). This connection allows us to provide reconstructions of genomes we postulate to encode some of the ‘most wanted' 16S rRNA gene sequences identified by Fodor *et al.* (2012) in the HMP data.

Briefly, our approach relies on the signal provided by the variation in the abundance of genomic entities across multiple metagenomic samples, under the assumption that genomic segments derived from a same organism exhibit a strong correlation in their abundance profiles. Such an approach has been successfully used to summarize metagenomic catalogs into coabundance gene groups and metagenomic species (MGS)—clusters of genes with correlated abundance profiles (Qin *et al.*, 2012; Le Chatelier *et al.*, 2013; Nielsen *et al.*, 2014). We cannot, of course, exclude the confounding effect of coassociations between unrelated organisms (e.g., symbiotic relationships between bacteria); however, we hypothesize that these correlations are weaker than those between genomic elements belonging to a same organism.

## Materials and methods

### Genus-level classification of OTUs

The databases SILVA, NCBI nt and NCBI WGS (Whole-Genome Shotgun) were used to retrieve the genus annotation for the OTU validation set. For the most wanted OTUs, we also relied on the RDP taxonomical annotation from Fodor *et al.* (2012). A threshold of 98% identity and 98% alignment coverage were used to define the genus annotation level in the different databases. For the RDP annotation, we only considered genera with classification confidence ⩾80%, and discarded any assignments that disagreed with the information from other databases.

### OTU data

The metadata and sequences for the most wanted OTUs (priority group, max fraction body habitat, RDP assignment and so on) were downloaded from the HMP website at http://www.hmpdacc.org/most_wanted/. The sequences and Qiime-processed (Caporaso *et al.*, 2010) abundance table for the HMP OTUs were downloaded from http://hmpdacc.org/HMQCP/.

### MGS genome metadata

The MGS metadata and sequences (genes, proteins, draft genomes) were downloaded from https://www.cbs.dtu.dk/projects/CAG/. Note that the MGS genomes were reconstructed from metagenomic data using a coabundance clustering approach similar to the one described here. They were further refined through sample-specific reassembly and the resulting assemblies were validated for completeness and correctness. For more details see Nielsen *et al.* (2014).

### Comparison between the closest NCBI references and MGS genomes

We compared the MGS draft genomes to genomes from NCBI database using the *dnadiff* tool from the mummer (Kurtz *et al.*, 2004) package with default parameters.

### Functional annotation

A functional annotation of the MGS proteins to the eggNOG groups (COG) was performed in two steps. First, the MGS proteins were assigned to the eggNOG 3.0 database with blastp (v.2.2.29+), using an e-value threshold of 1E−5. The first 20 hits for each protein were then considered for pairwise alignment to the MGS proteins using Clustal Omega (Sievers *et al.*, 2011). The sequence identity and coverage was extracted from the alignment. Finally, the COG was assigned from the hit that shared the highest sequence identity and coverage.

The PICRUSt COG prediction was performed with picrust-1.0.0 (Langille *et al.*, 2013) according to the authors' recipe for metagenome prediction.

### WGS mapping to genes

The reads of the 138 HMP samples were aligned to the MGS gene catalog 3.9M from the paper by Nielsen *et al.* (2014). The reads were mapped using Bowtie (Langmead *et al.*, 2009), by aligning the first 35 nt of the read and allowing up to three mismatches, and using the best-hit option.

### Translation to MGS and downsizing procedure

Raw count data were preprocessed to remove technical variability due to sequencing depth. We performed a downsampling procedure for each of the 138 samples by randomly selecting 15 million mappable reads and aligning them back to the reference catalog of 3.9 M genes (Nielsen *et al.*, 2014). The downsized data matrix was further normalized and transformed in relative abundance frequency using the RPKM+TC method. Specifically, each count was divided by the gene length and the total sum of the signal for each sample.

Genes from each MGS were ordered by decreasing connectivity (gene coabundance network based on Spearman's correlation and a threshold of 0.7). The 50 most connected genes were selected and used to compute the mean tracer vector for each MGS. This reduced data set was used for further analyses. These transformations were performed using the *momr* package (Prifti and Le Chatelier, 2014).

### ‘Validation set' and ‘most wanted' OTU abundance table creation

V3–V5 16S rRNA abundance table and taxonomic annotations were downloaded in a Qiime (Caporaso *et al.*, 2010)-formatted spreadsheet from: http://downloads.hmpdacc.org/data/HMQCP/otu_table_psn_v35.txt.gz.

The abundance table for the HMP most wanted OTUs was generated by mapping the shotgun sequences generated in the HMP project to the consensus sequences of the OTUs using Bowtie (Langmead *et al.*, 2009) with parameters ‘–q–v 1–k 1—suppress 1, 2, 4, 5, 6, 7, 8'. The resulting alignments were converted into an abundance table using custom software available from http://www.cbcb.umd.edu/software/MGS_correlation/.

### Correlation analysis for linking OTUs to MGS sequences

Correlation analysis was performed in R using custom software available from http://www.cbcb.umd.edu/software/MGS_correlation/. Briefly, abundance tables for OTUs and MGSs were normalized by conversion to ratios and were log-transformed to control variation and reduce the effect of extreme values. A pseudocount of 1E−17 was added to avoid underflow. For every MGS or most wanted OTU, we identified the OTU or MGS, respectively, whose abundance profile correlated best in terms of the Pearson's correlation value. We used Pearson's correlation to be consistent with the procedure used to construct the MGS clusters. Initial benchmarks using other measures of correlation revealed that the choice of measure did not significantly impact the results (data not shown). We focused our analysis on MGSs and OTUs that had positive counts in more than 10 samples (out of the 138 samples analyzed) to avoid spurious associations.

### Linking most wanted OTU and MGS genomes to association studies

The most wanted 16S rRNA sequences were aligned with BLAST against the Greengenes OTUs from the study by Goodrich *et al.* (2014) and were defined as similar only when the corresponding sequences shared more than 98% identity over 100% of the length of the most wanted sequence.

The MGS genomes were linked to gene clusters associated with health conditions by aligning the genes clusters to the MGS genes, and were defined as similar when at least 70% of the genes from a cluster matched the same MGS.

## Results

We derived a gene abundance table by mapping the shotgun reads from 138 stool samples sequenced in the HMP project (Human Microbiome Project C, 2012) to the 3.9M gene catalog constructed in the MetaHIT project (Qin *et al.*, 2010). These gene-level abundances were used to estimate the abundance of 741 MGS. We similarly derived abundance tables for 45 411 OTUs generated by the HMP from the V3–V5 hypervariable region of the 16S rRNA gene. We then identified correspondences between the metagenomic species and 16S rRNA OTUs by computing the correlation between the corresponding abundance profiles. We focused on the MGS correlated to 1468 OTUs (‘most wanted' OTUs) representing 16S rRNA gene sequences that were poorly characterized in public databases, but prevalent in the HMP human samples (Fodor *et al.*, 2012). While the original paper referred to only a subset of high-priority OTUs as ‘most wanted', for simplicity we will refer to all the sequences described by Fodor *et al.* (2012) as the most wanted set.

### Validation of our approach using taxonomically characterized OTUs

To validate our approach, parametrize it and evaluate its overall effectiveness, we used the full set of 45 411 OTUs generated by the HMP from the V3–V5 hypervariable region of the 16S rRNA gene and a subset of 387 high-quality MGS sequences. For each of the MGS sequences, we selected the best correlated OTU and estimated the accuracy of the association by comparing the genus-level annotation of the MGS and the OTU. Of the 387 best correlated OTUs, 104 had been annotated at the genus level, and 81 of these (78%) matched the genus-level taxonomic annotation of the corresponding MGS (see Supplementary Table 1, sheet OTU validation set). Manual inspection of the differences reveals that the majority of the disagreements involve OTUs assigned to members of the *Lachnospiraceae* family (Supplementary Figure 1), organisms that often confound automatic classification (Patil *et al.*, 2012). Many of the discrepancies may, thus, be artifactual. A Pearson's correlation coefficient cutoff of 0.650 was selected to balance the tradeoff between accuracy and error, in terms of the agreement between the genus labels of the MGS and OTUs as exemplified in Supplementary Figure 1.

### Linking the ‘most wanted' OTUs to genes and genomes

The ‘most wanted' OTUs identified by Fodor *et al.* (2012) are 16S rRNA sequences that are prevalent and abundant within the HMP samples but for which the corresponding genomes have yet to be sequenced. For each of the OTUs reported by Fodor *et al.* (2012) we retrieved the MGS sequence to which it best correlates in abundance, using a Pearson's correlation coefficient cutoff of 0.65 determined as described above. Among the 1468 OTUs, 201 (14%) could be associated to an MGS, thereby providing for the first time a genomic context for these OTU sequences. Of note, the majority (80%) of the ‘most wanted' OTUs that were assigned to an MGS by our coabundance method were primarily found in stool samples—unsurprising given that the MGS genomes were all reconstructed from stool. Another 15% were abundant in oral samples consistent with the well-documented transit of oral microbes through the digestive tract (Segata *et al.*, 2012). Among the stool OTUs considered ‘high priority' by Fodor *et al.* (2012), we were able to identify a corresponding MGS for half (23 out of 45, see Table 1).

In our selection procedure we only retained the most highly correlated MGS for each 'most wanted' OTU. The second best hit generally exhibited a lower correlation value, dropping from a median of 0.75 for the best hit to just 0.61 (below our correlation cutoff) for the second hit (see Supplementary Figure 2). For 134 OTUs (66% of the total set), both the first and second hits exceeded the 0.65 correlation cutoff. Genus-level annotations were only available for the MGS associated with 116 of these OTUs. In the vast majority of these cases (102/116, 88%), the genus-level annotations of the first and best hit was the same. The majority of the 14 disagreements in taxonomic placement between the first and second hits were between the genus *Faecalibacterium* and genera *Eubacterium* or *Ruminococcus*, disagreements that are likely due to the inconsistent taxonomic labels assigned to organisms within the Clostridiales order (see Supplementary Table S4).

To further validate the specificity of the approach, we attempted to identify fragments of the 16S rRNA gene within each MGS and compare them with the sequence of the 'most wanted' OTU. Owing to the difficulty inherent in assembling the 16S rRNA gene, we could only identify it in 19 of the MGS identified by our procedure, and in 18 cases, the metagenomically derived 16S rRNA sequence matched closely the OTU sequence associated with the corresponding MGS (see Supplementary Document 1, Section 1c). Furthermore, we note that 67 out of the 104 MGS sequences retrieved by correlation with the 'most wanted' OTUs (64%) could be correlated with both a 'most wanted' OTU and an OTU from the full HMP set (OTUs used in the validation described above). In 40 out of these cases (59% of the ambiguous cases and 38% of the MGS), an OTU from the full OTU set had a higher correlation value than the 'most wanted' OTU linked with the MGS. Having multiple OTUs match a same MGS is not surprising, given that the 16S rRNA gene is a multicopy gene. To verify whether the two OTUs assigned to a same MGS are related, we focused on the 28 'most wanted' OTUs corresponding to the V3–V5 hypervariable regions of the 16S rRNA sequence and compared them with the matching sequence from the full OTU set. Among these pairs, 21 (75%) matched each other with >97.5% identity as determined by BLAST (see Supplementary Table 1 and Supplementary Document 1, Section 1d).

### Genomes identified through correlation are highly concordant with sequences derived through advanced culturing techniques

**Figure 1:**
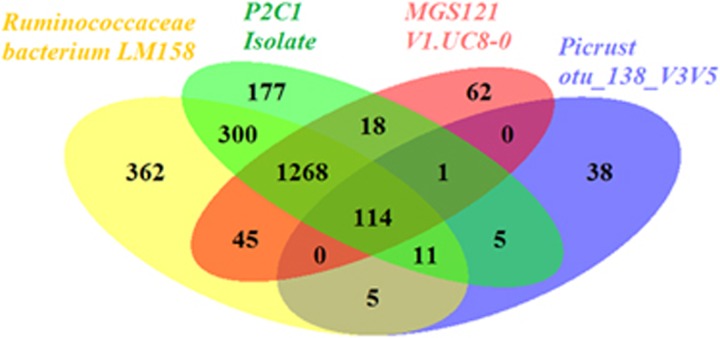
Comparison of functional content of genomes reconstructed by us, isolated through advanced culturing techniques and PICRUSt predictions. Numbers represent COG functional categories shared between MGS121—genome identified by our correlation-based approach; Ruminococcaceae bacterium LM158 and Oscillibacter-like P2C1 isolate—genomes isolated through advanced experimental techniques; and the PICRUSt prediction based on the sequence of otu_138_V3V5.

Several organisms related to most wanted OTUs were recently isolated and sequenced through advanced experimental technologies: (i) Ruminococcaceae bacterium LM158 (also called microfluidicus 1), isolated from a healthy American patient's cecum through a gene-targeted microfluidic cultivation approach (Ma *et al.*, 2014), (ii) *Oscillibacter*-like P2C1 isolate, sequenced from a healthy Danish patient's stool through a multiplex phenotyping cultivation approach (Rettedal *et al.*, 2014). These bacteria were selected by the authors of the respective studies due to the proximity of their 16S rRNA with the *Oscillibacter* genus, which is prevalent among the most wanted OTU sequences. Among these, otu_138_V3V5 shares 99.5% identity with the 16S rRNA sequence of the Ruminococcaceae bacterium LM158 and *Oscillibacter*-like P2C1 isolate. We found otu_138_V3V5 to be strongly correlated with the metagenomic species MGS121 (Pearson's correlation=0.84), a genome reconstructed from the MetaHIT sample V1.UC8-0 obtained from the stool of a healthy Spanish patient. To further explore the relation between these bacteria, we compared the functional composition of MGS121 V1.UC8-0 to the recently sequenced Ruminococcaceae bacterium LM158 and P2C1 isolates, as well as to the PICRUSt (Langille *et al.*, 2013) prediction of the functional composition of otu_138_V3V5 based on the sequence of the OTU alone (Figure 1).

The genome retrieved through our correlation-based approach, MGS121, shares most of the functional content with the two genomes reconstructed through complex isolation/culturing techniques (Figure 1). The PICRUSt prediction based on the sequence of the most wanted OTU only recovers a small fraction of the functional profile of this genome, highlighting the limits of prediction based on 16S rRNA sequences for organisms poorly represented in public genome database.

To further demonstrate that the bioinformatically identified genome sequences capture valuable biological information, we compared the MGS sequences we found to be associated with the most wanted OTUs to related genome sequences from public databases. Specifically, we identified 58 genomes containing 16S rRNA genes highly similar to those of ‘most wanted' OTUs (with ⩾98% identity over 100% length, see Supplementary Table 2). These 58 genomes were mainly isolated and sequenced by the HMP project and through another advanced culturing technique called culturomics (Lagier *et al.*, 2012) On average, when excluding five genomes from the Lachnospiraceae family, the MGS sequences shared high similarity (95.39% identity) to the publicly available sequences over a significant fraction of their length (83.76%). The accuracy was much worse for five genomes from the Lachnospiraceae family with 60.40% identity over just 25.64% of the length, likely due to insufficient resolution of the 16S rRNA gene sequence but we cannot exclude inherent limitations of our method within certain difficult taxonomic groups.

### A link between 16S rRNA and whole-metagenome-based microbiota-wide association studies

**Figure 2:**
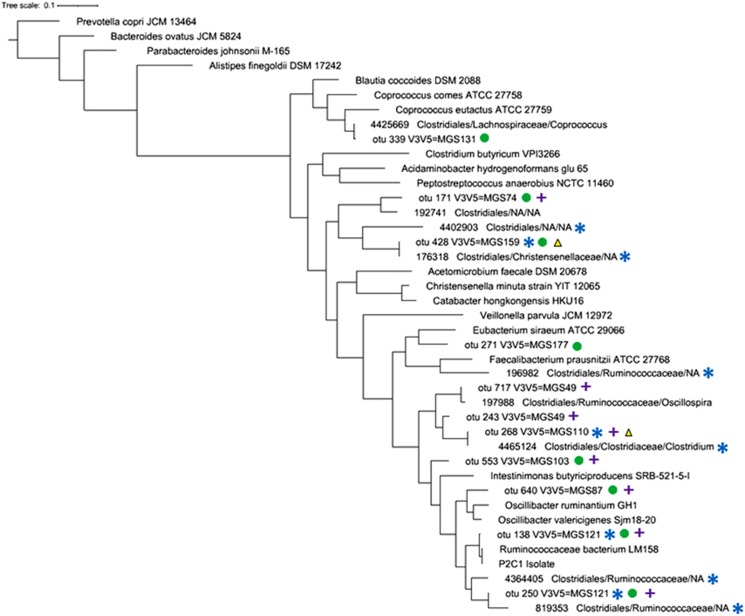
Phylogenetic tree (constructed with FastTree with default parameters) containing OTUs and MGS found to be significantly associated with health and host genetics. The colors refer to different association studies: (circles) Le Chatelier *et al.* (2013), (triangles) Cotillard *et al.* (2013), (crosses) Qin *et al.* (2014), (stars) Goodrich *et al.* (2014). The integer ID followed by taxonomical annotation are the Greengenes prokMSA IDs reported in the Goodrich *et al.* (2014) study. The closest NCBI references were added in the tree to provide a taxonomic context.

A recent study (Goodrich *et al.*, 2014) identified several 16S rRNA OTUs that are strongly associated with host genetic factors in United Kingdom and other countries. Several of the most wanted OTUs for which we could identify a corresponding MGS are highly similar to these sequences (Figure 2). We analyzed the MGS sequences that we could associate with these OTUs across several whole-metagenome studies, including two studies linking the richness of the gut microbiome with healthy outcomes in terms of metabolism within patients in Denmark and France (Cotillard *et al.*, 2013; Le Chatelier *et al.*, 2013), and a study of the gut microbiome in liver cirrhosis in China (Qin *et al.*, 2014). Across all these studies, a large number of MGS correlated to a ‘most wanted OTU' were observed as significantly associated with health in at least one study (see Materials and methods and Supplementary Table 3). Several of the MGS sequences were found to be associated with health in multiple studies or across multiple cohorts (Figure 2), including MGS121 that we discussed above.

A functional analysis of the MGS sequences we identified as correlated with these OTUs reveals several insights into their possible role within the gut ecosystem. All of the genomic sequences contain genes related to anaerobic cellular processes—unsurprising given that the healthy gut is believed to be populated by strictly anaerobic organisms, with the ecological balance shifting towards facultative anaerobes in disease (Walker and Lawley, 2013). Several organisms appear able to produce cobalamin (MGS74, MGS110, MGS121, MGS147, see Supplementary Figure 3) and butyrate (MGS159, MGS110, MGS49, MGS131), functions that are believed to be important contributions of the gut microbiota to human health. The human body cannot synthesize cobalamin, which is primarily contributed by the gut microflora, whereas butyrate is an important nutrient for human intestinal cells and it is believed to have a role in human health (Kau *et al.*, 2011; Flint *et al.*, 2015). One of the organisms found most associated with host genetic factors by Goodrich *et al.* (2014), prokMSA OTU 176318 linked by us to MGS159, appears to be involved in butyrate production and also contains genes related to nisin resistance. Nisin is a broad-spectrum bacteriocin produced by lactic acid bacteria, and likely has a role in the probiotic properties of these organisms (Pessione, 2012). The fact that OTU176318/MGS159 is resistant to nisin may be consistent with it being associated with a healthy gut.

The above-mentioned MGS121 contains a γ-aminobutyrate permease, possibly indicating this organism may be able to synthesize or metabolize this compound. The biosynthesis of γ-aminobutyrate also has relevance to human health as this compound has beneficial properties—it has been shown to control blood pressure and appetite, and has also been implicated in the purported connection between the gut microbiota and the host central nervous system (Hemarajata and Versalovic, 2013). Finally, MGS131 appears to contain multiple genes from the VanB vancomycin resistance operon suggesting the possibility of using targeted culturing approaches for isolating this organism, similar to the approach used to isolate the P2C1 strain discussed above (Rettedal *et al.*, 2014).

## Discussion

The increased availability of metagenomic data sets spanning hundreds of samples provides an unprecedented opportunity for the development of novel comparative methodologies. Nielsen *et al.* (2014) have recently shown that the abundance profile of metagenomic genes across multiple samples can be used to organize the data into metagenomic units, representing clusters of covarying genes. Here we have leveraged this information to provide a first correspondence between 16S rRNA-based and whole-metagenome surveys of the gut microflora, as well as to link the information constructed in the European MetaHIT project with that generated in the US NIH-led HMP project.

When applied to a collection of 16S rRNA OTUs determined by the HMP to be ‘most wanted' because of their high prevalence in the HMP samples and their relative absence from public databases, our method was able to identify corresponding MGS sequences for a large number of OTUs, including roughly half of the high-priority organisms from the human stool. We are thus able to provide a first genomic and functional context for a number of ‘most wanted' organisms, complementing current efforts aimed at isolating and sequencing these organisms through costly culture and single-cell-based experimental strategies. Comparison of the genomes we identified through correlation with experimentally derived genomes reveals high sequence identity and concordance in predicted function.

Analysis of several of the most wanted OTUs across multiple association studies, based on both 16S rRNA and whole-metagenome sequencing, found these organisms to be consistently associated with health—unsurprising given that the HMP project focused on healthy individuals. Many of these organisms are prevalent across studies and continents, yet most have yet to be isolated and sequenced. These findings suggest the need for a better characterization of the organisms found in the healthy human microbiome, some of which may be important for maintaining health.

These results indicate that valuable biological insights can be obtained through data integration across multiple experimental platforms, despite experimental limitations such as PCR amplification biases and other technical challenges (both experimental and bioinformatic). At the same time, our study was hampered by ambiguous and incorrect taxonomic labels in public databases, particularly within the *Lachnospiraceae* family, underscoring the need for further development and refinement of taxonomic databases, particularly keeping in mind the need of large-scale automated computational analyses of the data.

Despite our initial success, it is important to also note several limitations of the approach used. As already mentioned, spurious correlations may occur between 16S rRNA gene sequences, and, thus, the results we obtain are simply hypotheses to be further explored rather than the absolute truth. We also note that for a same 'most wanted' OTUs we could often identify multiple MGS sequences with a correlation value higher than our selected cutoff of 0.65. In the vast majority of cases, the multiple hits had the same genus-level taxonomic classification. This observation may imply that multiple closely related organisms inhabiting the same ecological niche have similar abundance profiles. As such, these organisms are difficult to distinguish from each other by coabundance methods and may confound our approach. Larger numbers of phenotypically different samples will likely reduce this confounding effect as different organisms, even if closely related, will respond differently to environmental parameters. We also recommend that in such situations researchers pursue in further validation experiments not just the best hit, but all the MGS sufficiently well correlated with an OTU.

Furthermore, it is important to note that the MGS genomes we linked to the 'most wanted' 16S rRNA gene sequences were themselves reconstructed through coabundance techniques and likely capture just the core genome of the corresponding organisms. The accessory genes within the pangenome frequently have patterns of abundance different from those of the core genome, and may not, thus, be detected through coabundance-based approaches. As such, they represent just a partial reconstruction of the 'most wanted' genomes. Nonetheless, the availability of even a partial genomic reconstruction is valuable. This sequence can be used to design probes for the targeted capture of DNA from the organism of interest, and the genes contained within can provide insights into possible strategies for isolating the corresponding organism (e.g., by supplementing growth media with necessary nutrients or by inhibition of competing organisms through antibiotic treatment).

Our study and parallel efforts in the community (Carr *et al.*, 2013; Alneberg *et al.*, 2014) reveal the tremendous power provided by cross-sample analyses. The information contained in the abundance profiles of genes and genomic entities across multiple samples with differing characteristics can be viewed as a proxy for the phenotypic properties of individual organisms, information that can currently only be determined for culturable organisms. We note that this is one example where metagenomic experiments comprising multiple samples provide more information than traditional genomic studies on isolate genomes, and hope our work will spur the development of new approaches that can leverage such data in the characterization of the majority of microorganisms that cannot be currently isolated and grown in culture.

## Figures and Tables

**Table 1:** Summary of ‘most wanted' OTUs and correspondence with MGS stratified by body site and importance^a^

*Priority level as defined by Fodor et al. (2012)*	*Dominant human body habitat*	*Number of ‘most wanted' OTUs*	*‘Most wanted' OTUs with a corresponding MGS*
High priority	Total	119	24 (20.17%)
	Stool	45	23 (51.11%)
	Oral	64	1 (0.02%)
	Other	10	0
			
Medium priority	Total	338	49 (14.5%)
	Stool	127	42 (33.07%)
	Oral	176	5 (2.84%)
	Other	35	2 (5.71%)
			
Low priority	Total	1011	128 (12.66%)
	Stool	313	96 (30.67%)
	Oral	445	24 (5.39%)
	Other	253	8 (3.16%)

Abbreviations: HMP, Human Microbiome Project; MGS, metagenomic species; OTU, Operational Taxonomic Unit.

a Importance is defined by Fodor *et al.* (2012). ‘High priority': <90% identity to either the GOLD-Human or HMP strains database. ‘Medium priority': HMP OTUs with >90% identity and <98% identity to GOLD-Human or HMP strains database were assigned to a ‘Medium priority' group. ‘Low priority': HMP OTUs with >98% identity to either the GOLD-Human or HMP strains database.
