# Supplementary material for: Capturing the most wanted taxa through cross-sample correlations
Source: ISME J. 2016 Mar 4;10(10):2459–67. doi: 10.1038/ismej.2016.35 (PMC5030688; doi:10.1038/ismej.2016.35)
Supplement: Supplementary Figures [file ismej201635x1.doc]

# Validation of co-abundance approach

## Comparison of MGS versus OTU taxonomy


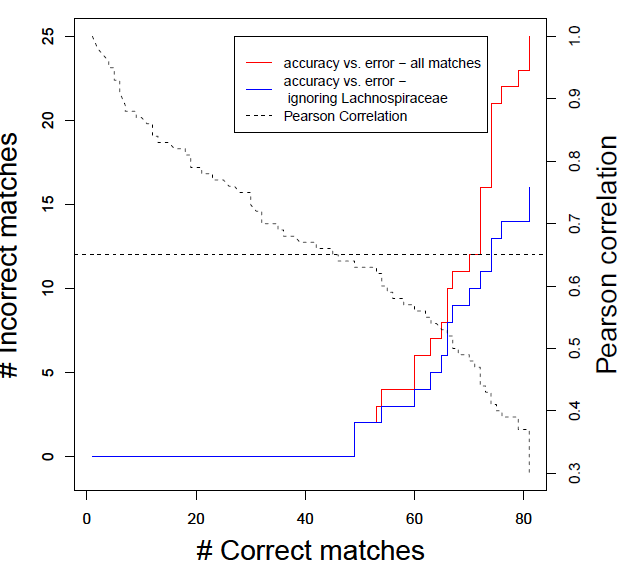


Supplementary Figure 1. Tradeoff between accuracy and error when varying the correlation threshold. The red line highlights the tradeoff between accuracy and error for all OTUs. The blue line excludes discrepancies within the Lachnospiraceae family. The dashed line represents the Pearson correlation coefficient corresponding to the data presented in the solid lines. The inflection point, where the number of errors increases faster than the number of correct assignments corresponds to a Pearson correlation coefficient of 0.63. x-axis and left y axis report the number of OTUs matching and mismatching in taxonomy, respectively (the solid lines in the graph). The right y axis represents the Pearson correlation coefficient (the dashed line in the graph).

## Comparison between first and second best correlated hits


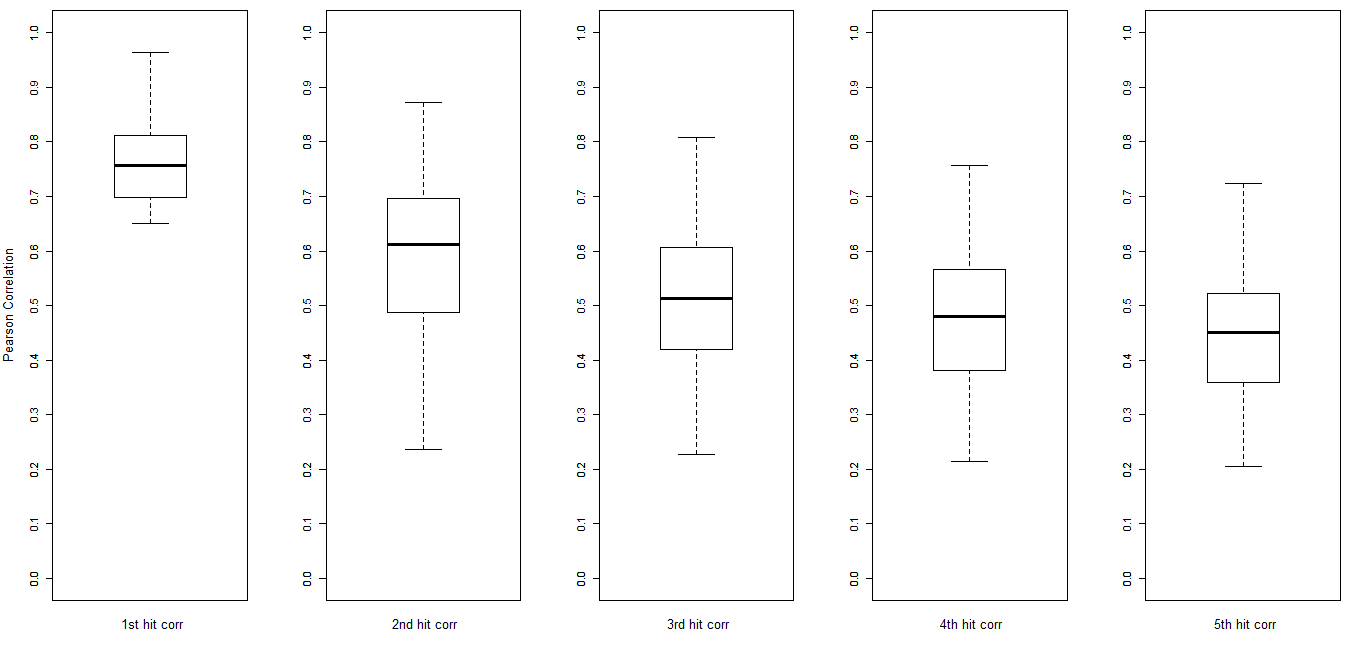


Supplementary Figure 2. The Pearson correlation distribution for the first through fifth best hits when recruiting MGS sequences against 'most wanted' OTU sequences. A large drop in Pearson correlation is apparent between the first and second hits.

## Agreement between OTU identified by co-abundance and 16S rRNA gene fragment within MGS

We aligned, using BLAST, the most wanted OTUs to the MGS genome retrieved by co-abundance in order to determine whether fragments of the 16S rRNA gene exist within the MGS and whether they agree with the OTU sequence. We could only identify 19 MGS that contained sufficiently long (>200bp) fragments of the 16S rRNA gene. In 18 of these cases the most wanted OTU agreed in sequence (>200bp, > 98% identity) with the MGS sequence.

## Agreement of Most Wanted OTUs and QIIME OTUs matched to a same MGS

We compared the results of our validation experiment (MGS sequences correlated to the complete set of HMP OTUs) with the results obtained for the 'most wanted' OTUs in order to determine whether a same MGS sequence was found to be correlated with both a 'most wanted' OTU and an OTU from the full set. We only retained the 'most wanted' OTU that was best correlated with the MGS in the case where multiple OTUs matched a same sequence. Among these sequences we focused on just those for which an OTU from the full set had a higher correlation value than the 'most wanted' sequence, and further analyzed just the sequences derived from the V3-V5 hypervariable regions of the 16S rRNA gene so that we could directly compare them to the V3-V5 OTUs used in our validation. For each pair of OTUs, one from the 'most wanted' set and one from the 'validation' set, we retrieved the corresponding sequences and performed a pairwise comparison using BLAST.

# Cobalamin synthesis pathways found in multiple MGS


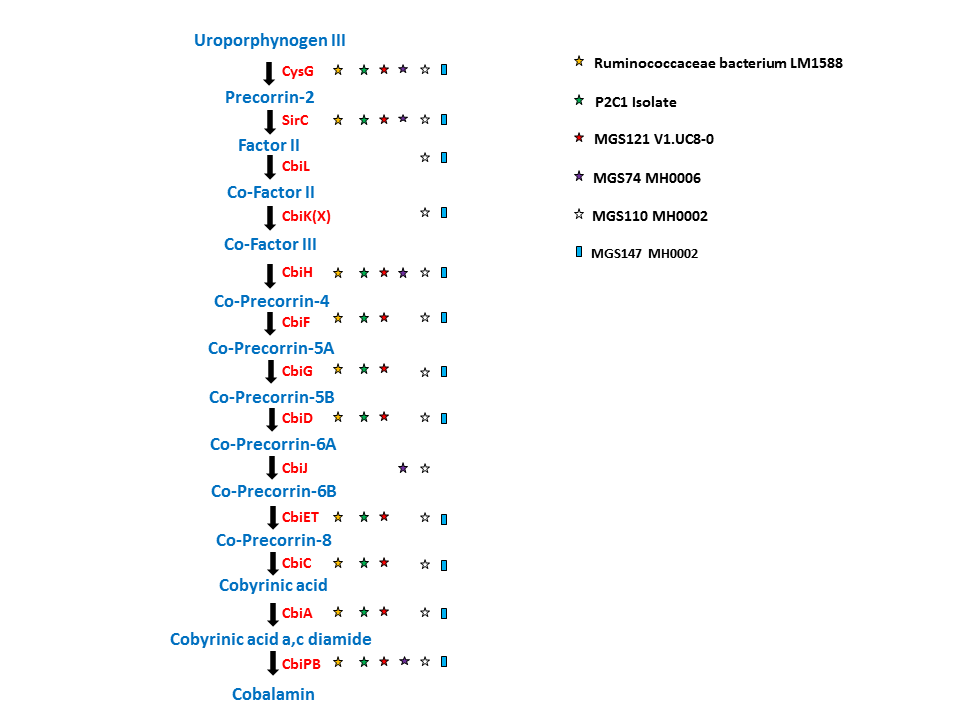


Supplementary Figure 3. Anaerobic pathway for cobalamin synthesis found in multiple MGS retrieved by our approach.
